# The Pathology of Gout

**Published:** 1906-03-31

**Authors:** 


### Hospital Clinics.

### THE PATHOLOGY OF GOUT.

A review of the present position of knowledge in the matter of the pathology of gout appears in the February issue of the "London Hospital Gazette," from the pen of Mr. H. L. Tidy. "Since the days of Sydenham," he says, "the pathology of gout has been a favourite question in the dialectics of medicine. The essential points which are undeniable are the presence of an excess of uric acid in the blood, and the deposition of sodium biurate in the articular and other tissues of gouty individuals. No other point is beyond discussion, and neither of these is pathognomonic of gout." At the present time many observers are prepared to see in creatinine and such substances the real cause of the deposits in gout. Mr. Tidy's remarks are, however, confined to the purin bodies.

The purin bodies, of which so much is heard in these days of higher dietetics, bodies, for example, such as uric acid, xanthin and hypoxanthin, owe their relationship of a common skeleton, namely the ring called the "purin nucleus" and shown in the accompanying structural formulæ:—

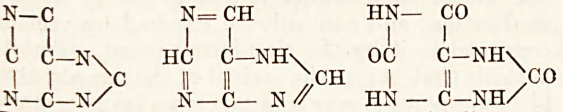

Purin nucleus. Purin. Uric acid.

Uric acid was discovered in 1776 by Scheele, who isolated it from the urine. In 1787 Woollaston showed that the deposits in gouty tissues consisted of urates. In 1847 Sir Alfred Garrod detected urates in the blood of a gouty individual, and this presence has since been proved to be constant. Uric acid is very closely allied to urea, two molecules of the latter being obtainable by a process of oxidation and hydrolysis from one molecule of uric acid, while conversely uric acid may be produced by the condensation of urea with hydroxy acids.

The accumulation of uric acid in the blood in the gouty state is possible of explanation on three hypotheses. Firstly, that there is an over-production at the ordinary sites of uric-acid formation, this excessive production being accompanied by a normal excretion. Secondly, that the formation of uric acid is normal in amount, but that its destruction in the ordinary course is diminished. Thirdly, that the formation of uric acid is, again, normal in amount, but the excretory power of the kidneys is deficient. Now over-production can be imitated by the subcutaneous injection of uric acid, while physiological over-production occurs after the ingestion of great quantities of the purin bodies, but in either case, so long as the kidneys are healthy, this over-production is accompanied by an increased secretion, showing that normal kidneys are capable of dealing with more than the usual bulk of uric acid. Therefore, if in gout there is an excessive production of uric acid we should expect to find an increase in the excretion of this substance, but we do not. This last fact serves also to eliminate the second hypothesis, namely that a normal formation of uric acid is in gout accompanied by a diminished destruction. *It appears, therefore, that deficient excretion by the kidneys is the only hypothesis compatible with the observed phenomena of gout.* Direct determinations support this thesis, for the excretion of uric acid by gouty patients is distinctly below that of healthy individuals on a similar diet. Retention, therefore, is the explanation of the excess of urates in the blood of gouty people.

Now, since paroxysms of gout never occur when urates are absent from the blood, it is obviously of importance to recognise their source, in order to cut off the supply if possible. What then is the origin of urates and purin bodies? There are again three possible origins for the purin bodies found in the urine: 1. Synthetic formation within the organism. 2. Exogenous origin, these bodies being elements of the diet. 3. Endogenous origin, from katabolism of tissues. Firstly then of synthesis. In birds, a synthesis of uric acid undoubtedly takes place. The administration of urea to birds results in an increased excretion of uric acid. In the case of mammals the purin excretion remains almost constant on a purin-free diet, but a few chemical substances which are not purins do cause a slight but perceptible increase in the purin excretion of this class. It is probable, therefore, that a small proportion of the urates excreted by mammals has a synthetic origin.

Secondly, as regards the exogenous hypothesis. Twenty years ago it was believed that the metabolism of any proteid gave rise to uric acid, and that urea and uric acid had a common source. This is now known to be an error, for on a flesh-free diet the uric-acid excretion remains constant though the urea excretion rises and falls with the quantity of proteid ingested. It is recognised that only certain foodstuffs are capable of raising the excretion of purins. Of these there are three groups:—

1. Oxy-purins, such as xanthin and hypoxanthin which are present in *muscle and meat-extracts.*

2. Amido-purins. It has been found that certain animal foodstuffs such as thymus gland and pancreas, which in simple extract contain but negligible quantities of xanthin bases, are yet capable of increasing the excretion of purins. This capacity has been shown to depend upon the metabolism of the nucleins contained in these substances. *All nuclein-containing foodstuffs influence the purin-excretion.*

3. Methyl-purins. Such bodies as caffeine and theobromine raise the total of purin excretion, but do not influence the excretion of uric acid.

Thirdly, touching the endogenous hypothesis. The fact that on a purin-free diet individuals continue to excrete purins, demonstrates the existence of an endogenous formation of these bodies, a formation, that is to say, depending upon metabolism of tissues. Now the purin-bodies contained in animal tissues are either amido-purins or oxy-purins, represented respectively by the thymus and pancreas on the one hand, and by muscle on the other. The destruction of leucocytes is a constant source of nuclein-disintegration, but it is not the sole source of endogenous purins. It has been found that muscular exercise largely increases the excretion of purins. Also, hypoxanthin has been found in defibrinated blood after perfusion through the hind legs of a dog the muscles of which have been thrown into tetanus.

The following conclusions of Burian and Schur, Folin, and others are of importance: (1) That the exogenous and endogenous moieties are mutually independent; (2) the exogenous moiety depends in amount solely on the diet; (3) the endogenous moiety varies with each individual, but for any given individual is constant irrespective of diet, given similar physiological conditions—*e.g.* of muscular exercise.

Now in chronic gout the endogenous purin excretion is on a par with the lowest average of healthy individuals, and while the administration of thymus is followed by an increased excretion, that increase is not so large as is the case in health. During an acute attack the excretion of uric acid falls to a low level, rises to the normal shortly after the onset of the paroxysm, and then again subsides.

Admitting that the urates in the blood are due to deficient excretion it remains to consider the two possible explanations of the defect. They are these: *either gout is a renal inadequacy, or the uric acid presented to the kidney of the gouty person is in some way abnormal.* In support of the renal theory it may be said that renal lesions are frequently associated with gout, and that urates are constantly present in the blood of sufferers from chronic interstitial nephritis. The form of the uric acid in gout remains in the domain of hypothesis; the author, however, considers that some abnormality in the uric acid is a likely contributory cause of the faulty excretion of the latter.

The primary cause of acute gout has been attributed to the presence of uric acid in the blood acting either as a toxic agent while in solution, or as an irritant crystalline compound. There is no evidence that uric acid is toxic in solution, but the bulk of opinion inclines to the explanation of Sir William Roberts, viz., that the gouty paroxysm depends upon the crystallisation of sodium biurate in the affected area, and a consequent mechanical irritation of the tissues in the vicinity. This authority held that the urates occurred in solution as the soluble quadriurates of sodium; these were gradually converted into biurates by the sodium carbonate of the blood, and in this form were deposited when the blood became saturated. The existence of these quadriurates can no longer be admitted. Diminished alkalinity of the blood does not affect the deposition of biurate of soda, as suggested by Haig. The deposit takes place almost exclusively in fibrous tissue structures, and especially about certain joints. Sir William Roberts has proved that the proportion of sodium salts present in the medium is an important factor in determining the deposition of biurates. He has also found that in the body the highest percentage of sodium salts occurs in cartilage, synovia, and fibrous tissue.

